# Incremental peritoneal dialysis after unplanned start initiation

**DOI:** 10.3389/fneph.2022.932562

**Published:** 2022-07-25

**Authors:** Viviane Calice-Silva, Fabiana Baggio Nerbass

**Affiliations:** ^1^ Nephrology Division, Pro-rim Foundation, Joinville, Brazil; ^2^ Medicine School, Universidade da Região de Joinville (Univille), Joinville, Brazil

**Keywords:** incremental dialysis, urgent-start peritoneal dialysis, unplanned peritoneal dialysis, peritoneal dialysis, early-start peritoneal dialysis

## Abstract

Incremental peritoneal dialysis (PD) is characterized as less than a “standard dose” PD prescription. Compared to standard treatment, it has many potential advantages, including better preservation of residual renal function, a lower risk of peritonitis, and a decreased care delivery burden while reducing the environmental impact and economic cost. Unplanned PD can be defined when treatment starts up to 14 days after catheter insertion and is recognized as a safe and feasible clinical approach. In this perspective paper, we briefly discuss both strategies and share our experience and clinical routine in managing incremental PD after unplanned initiation.

## Introduction

In the past, patients with end-stage kidney disease (ESKD) were meant to start renal replacement therapy (RRT) at the time their glomerular filtration rate (GFR) was reduced from 10 to 15 ml/min/m^2^ according to their comorbidities, starting dialysis earlier if diabetic or later if not diabetic (1). Also, most patients at their dialysis initiation were prescribed similar dialysis doses regardless of their residual kidney function (RKF) or needs, both in hemodialysis (HD) or peritoneal dialysis (PD) modalities. At that time, the lower dialysis dose prescribed was considered suboptimal and against the standard of care (2).

However, the identification of some dialysis-related complications associated with survival, such as infections, hemodynamic instability leading to ischemic complications loss of residual kidney function (RKF) have changed the clinical practices worldwide, favoring a slower dialysis initiation. This new approach preserves RKF, improves the quality of life of patients, treatment adherence, and outcomes (3).

Incremental PD is a strategy in which the dialysis dose is low at the beginning and progressively increases over time according to the reduction in RKF. Incident PD dialysis patients may have substantial residual kidney function, thus allowing less than a full dose of peritoneal dialysis, which may lead to fewer complications over time (2).

Another important point to be mentioned is that unplanned PD has increased the application of this modality to ESKD patients globally. Current data show the safety and viability of this modality compared with planned PD or unplanned hemodialysis (4). Those two strategies may complement each other because most patients starting unplanned PD have preserved renal function and may not need full-dose PD at the beginning of their treatment. In this article, we discuss the main aspects of both strategies and our perspective on how to implement incremental PD after unplanned PD initiation in clinical practice.

## Incremental peritoneal dialysis

The incremental PD definition varies in the literature. In 2020, Blake and colleagues proposed the following definition:

“Incremental PD is a strategy by which less than standard “full-dose” PD is prescribed in people initiating PD so that the combination of residual renal and peritoneal clearance achieved is sufficient to achieve individualized clearance goals; it is done with the intention of increasing the peritoneal prescription if and when residual renal clearance subsequently declines” (3).

In clinical practice, incremental continuous ambulatory peritoneal dialysis (CAPD) prescriptions are those with less than the typical daily “full-dose” dwells, dwell volumes, days-a-week treatment, or some combination of these. Incremental automated peritoneal dialysis (APD) prescriptions include either APD without a long dwell, less than 10 L daily delivered by cycler and day dwells, treatment for fewer days per week, or some combination of these (3).

Although not deeply explored, studies have showed that incremental PD has clinical benefits that add to the patient perspective and economic and environmental advantages compared with standard treatment (**Table 1**).

**Table 1:** Advantages of incremental peritoneal dialysis.

Clinical	–Slower decline in residual renal function (5) –Lower risk of peritonitis (6) –Decreases peritoneal glucose exposure, a risk factor for peritoneum failure (7) –Individualization of PD prescription, attending person- centered care principles (3)
Patient perspective	–More time for life participation (8) –Decrease the burden of care delivery (9) –Better quality of life (8)
Economic	–Reduce costs (8) –Reduce the risk of future solution shortage (9)
Environmental	Decrease the generation of nonrecyclable waste and carbon footprint (10)

As stated by Cheetham et al., these benefits need to be weighed against potential disadvantages. They include suboptimal dialysis, small solute clearance, fluid overload, effects on patient survival due to underdialysis, and the possible reluctance of patients to increase their PD prescription when indicated. “Shared decision-making, with discussion of advantages and disadvantages, may help alleviate patient resistance to necessary treatment changes. Both clinicians and patients must be mindful of the anticipated trajectory of PD treatment” (11).

Treatment adherence and adequacy need to be closely followed by a well-trained multidisciplinary team and proper infrastructure to maximize the benefits of this strategy. Patient education and dietary counseling can be especially important to maintain nutritional status, uremic toxins, electrolytes, and fluid status adequately (12).

The lack of studies comparing incremental and full-dose PD was highlighted by a recent systematic review and meta-analysis of cohort studies of adults in which only seven investigations met the inclusion criteria. They concluded that incremental dialysis allowed longer preservation of renal kidney function, delaying full-dose dialysis start by 12 months with no increase in mortality risk. Furthermore, no evidence of any harmful effect of incremental PD was observed (5).

## Unplanned peritoneal dialysis

Unplanned PD, also known as urgent-start PD (US-PD), was first defined as therapy initiation within 14 days of PD catheter insertion, since the International Society for Peritoneal Dialysis (ISPD) and European Renal Best Practice (ERBP) guidelines suggest a break-in period after catheter placement of at least 15 days (13, 14). Unplanned PD has gained more attention recently due to its favorable outcomes compared with planned PD or urgent start hemodialysis (4, 15). A recent systematic review and meta-analysis that evaluated the feasibility and safety of US-PD found no difference in mortality, peritonitis, exit-site infection, or PD technique survival compared with planned PD. However, a higher incidence of leakage and catheter mechanical dysfunction was observed in US-PD. Compared with US-HD, the all-cause mortality was similar, and bacteremia was significantly lower in the US-PD group (4).

Recently, Blake and Jain proposed two different terms to define unplanned PD: urgent-start PD and early-start PD. The first should be reserved for patients with genuinely urgent clinical presentations requiring PD within 72 h of catheter insertion. The more elective variant, where PD is started between 3 and 14 days after catheter insertion and may undergo hemodialysis (HD) before PD, is best termed “early-start PD” (16). Based on these new criteria, we compared the outcomes of 72 patients from our center who initiated unplanned PD (40 as urgent-start and 32 as early-start). They were similar regarding demographic characteristics, 30-day complications, 6-month hospitalization, and dropout events (17).

Since new PD patients need clinical compensation, equipment, and training to perform the dialysis at home, the first weeks of unplanned PD are conducted in the dialysis centers as intermittent peritoneal dialysis (IPD). The number of days a week, hours per session, and fill volume are individualized according to clinical signs and symptoms and also laboratory evaluation.

## Unplanned PD in our center

An unplanned PD program was implemented at our center in 2016. As a routine, after the nephrologist evaluation, lack of contraindication, and patient interest in the PD modality as renal replacement therapy, the nursing team evaluates the candidate. Around a fifth of patients referred to unplanned PD in our center are contraindicated by nursing due to self-care inability associated with the lack of family support (18).

Since 2016, almost 300 patients have initiated unplanned PD, more than half up to 72 h after catheter insertion. Besides allowing patients to choose between PD and HD when both treatments were indicated, the number of patients on PD increased by 150% and currently represents 30% of the dialysis patients in our clinic. According to a national survey, only 7.4% of chronic dialysis patients were on PD in Brazil in 2020 (19). Our outcomes in patients who started unplanned PD compared to planned PD were recently analyzed, and 30-day complications and first-year outcomes were similar in both groups (15).

## Unplanned PD followed by incremental PD in clinical practice

From our perspective, incremental PD is a strategy particularly interesting for patients who start PD urgently since many are not aware of CKD due to a lack of diagnosis and did not receive any predialysis care. Receiving a diagnosis of a chronic condition that profoundly affects lifestyle habits and routines such as end-stage kidney disease is challenging and commonly affects patient and caregivers mental health (20, 21).

As it is a home therapy in which the patient or a caregiver performs the treatment, the main challenges are faced initially. Therefore, less dialysis in terms of days and hours can decrease the burden of care and enable more life participation, a central goal of PD treatment according to patients (22).

Incremental PD is the preferred strategy to initiate patients at our center, regardless of whether PD was planned or not. After finalizing their training, those patients starting unplanned PD at our center move from in-center IPD to incremental PD or full-dose PD according to their clinical conditions, RKF, and toxin levels. Each patient undergoing IPD is evaluated weekly through blood routine workup, which includes hemoglobin, creatinine, urea, potassium, phosphate, albumin, and bicarbonate. According to these results, their PD prescription is adjusted to improve solute clearance and avoid clinical complications. Also, phosphate binders, diuretics, and other medications are prescribed if necessary. A multidisciplinary team also follows all patients to provide dietary, psychological, and social orientation and support.

**Figure 1:**
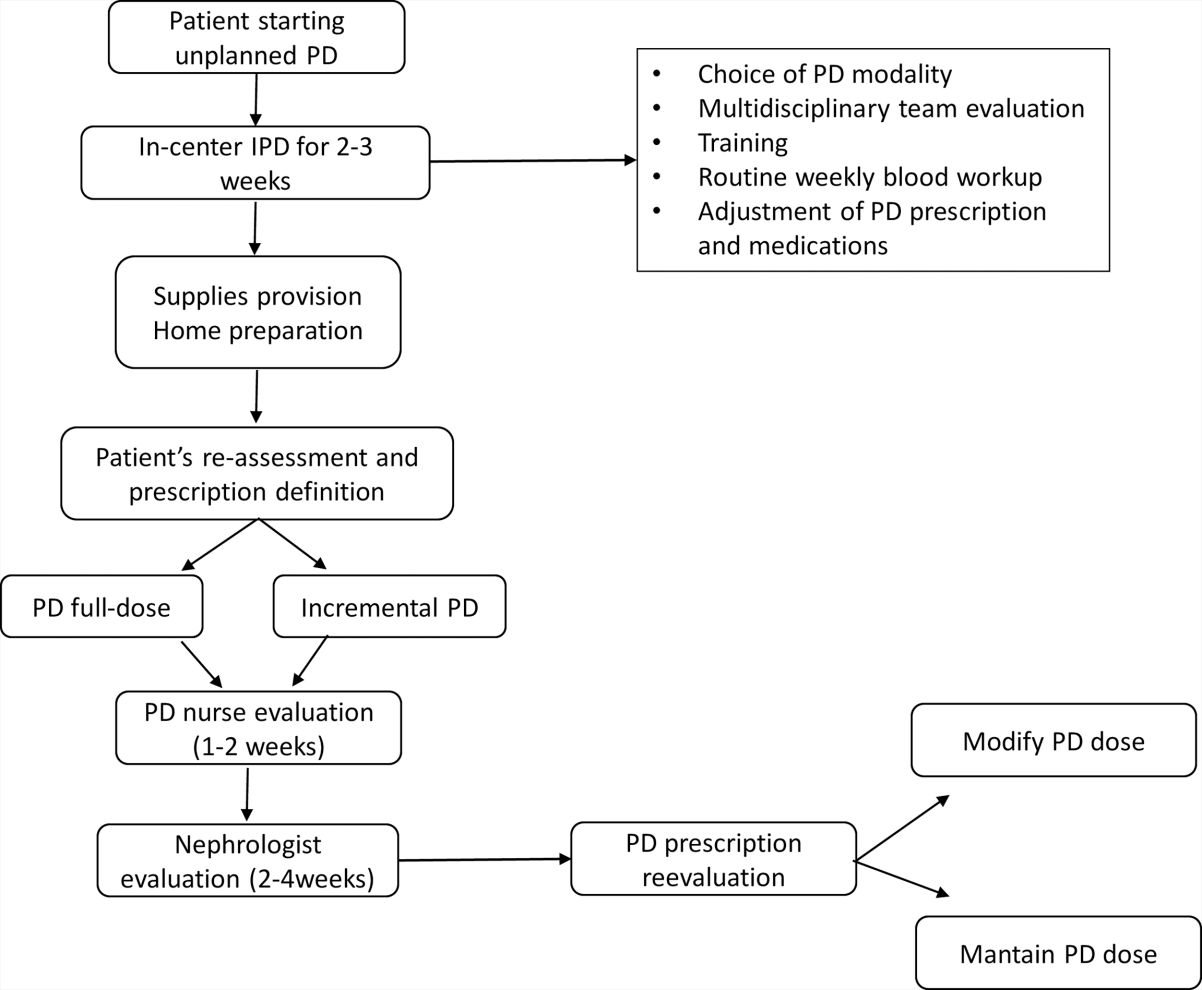
Clinical routine for unplanned peritoneal dialysis initiation. PD, peritoneal dialysis; IPD, intermittent peritoneal dialysis.

When the time comes to start PD at home, patients and caregivers are informed which prescription needs to be filled at home according to the current condition from the medical perspective. Nephrologists always consider the preferences of patients and caregivers when considering PD modalities and their daily home routine. Patients who eventually start full-dose PD are informed that the dialysis dose can be reduced according to the next laboratory results and the clinical condition. Some examples of clinical and laboratory parameters considered are blood urea nitrogen (BUN) (>200 mg/dl), potassium (>5.0 mEq/L) and phosphate levels (>5.5 mg/dl), as well as the presence of uremic symptoms or hypervolemia with current dialysis prescription and medications. After home PD initiation, patients return after two weeks for nurse evaluation and after 3-4 weeks for nephrologist consultation. From then on, monthly appointments are scheduled. When necessary, patients are evaluated more frequently (**Figure 1**).

Nowadays, around two-thirds of unplanned PD patients, after the training period, have a prescription for incremental dialysis. The ones that start with a full dose are reevaluated, and if RFK improves and/or laboratory parameters allow, the prescription is readjusted to an incremental strategy. Furthermore, to get patients and caregivers involved and conscious regarding the potential necessity of increasing dialysis doses in the future, they are constantly informed about the progression of kidney disease and the consequences of increased uremic toxins on their bodies.

Our patients usually stay on incremental PD for about 12 to 24 months after dialysis initiation. We assess clinical evaluation, standard laboratory work, and urine output monthly, and KT/V quarterly. When patients present signs of RKF reduction, not only demonstrated by urine output reduction but also by increase in uremic toxins level, potassium, and phosphate, additional strategies are tested instead of increasing dialysis dose to maintain patients in the incremental strategy for as long as possible. Again, nutritional counseling, medication adjustments, treatment adherence evaluations, and psychological interventions are offered when necessary. When interventions are not effective, the dialysis dose is increased.

## Discussion

In 2020, the International Society for Peritoneal Dialysis (ISPD) suggested in their PD practice guideline the use of innovative PD care delivery strategies in people initiating PD, potentially improving the experience of patients while lowering complication rates and costs (8). Both unplanned and incremental PD are closely aligned with ISPD recommendations.

These strategies may increase PD penetrance worldwide and allow more patients to receive RRT, especially in developing countries.

Besides the effort of nephrology teams to enable PD as an RRT option for most people, it also needs governmental incentives. In some countries, strategies that have led to increased use of PD have included the implementation of policies that allowed the production and supply of materials at a low cost and appropriate training for nephrology teams to increase the use of therapy and reduce failure rates (23). In Brazil, the main reason for the low PD use seems to be the model proposed by our public health system, which is not economically viable for most clinics (24).

While there is scientific evidence of the safety and favorable outcomes of unplanned PD, incremental PD remains uncertain due to the limited number, size, duration, and quality of studies performed (11). Only one randomized controlled trial (RCT) compared incremental to full-dose CAPD in 139 incident patients. After 24 months, three and four exchanges had similar effects on residual glomerular filtration rate, urine volume, and time to anuria. However, the incremental group had a longer peritonitis-free survival time (6). Large and well-conducted RCTs of standard *vs*. incremental, including patient-reported outcomes and economic feasibility concerning short- and long-term cost-effectiveness, are warranted.

Despite the lack of evidence, patients and caregivers seem to better accept and adapt to the treatment with the incremental strategy. Other advantages are that there is no need for additional support or a multidisciplinary team other than the regular ones in the day-by-day routine. The same team that takes care of planned and full-dose PD patients can easily follow incremental PD patients.

Meanwhile, based on our experience, we recommend using incremental PD. When well conducted by the nephrology team and under the preference of patients, this strategy provides high-quality care while reducing environmental impact and economic cost.

## Author contributions

Both authors contributed equally to the article considering conception or design of the work, drafting the work and revising it critically as well as provided approval for publication of the content and agreed to be accountable for all aspects of the work.

## Conflict of interest

The authors declare that the research was conducted in the absence of any commercial or financial relationships that could be construed as a potential conflict of interest.

## Publisher’s note

All claims expressed in this article are solely those of the authors and do not necessarily represent those of their affiliated organizations, or those of the publisher, the editors and the reviewers. Any product that may be evaluated in this article, or claim that may be made by its manufacturer, is not guaranteed or endorsed by the publisher.
